# The SHINE trial (a multicentre, randomised trial of stabilisation with nasal high flow during neonatal endotracheal intubation): statistical analysis plan

**DOI:** 10.1186/s13063-021-05390-7

**Published:** 2021-08-24

**Authors:** Kate Hodgson, Brett Manley, Omar Kamlin, Louise Owen, Calum Roberts, Kate Francis, Peter Davis, Susan Donath

**Affiliations:** 1 Newborn Research Centre, Royal Women’s Hospital, Level 7, 20 Flemington Rd, Parkville, Victoria 3052 Australia; 2 Department of Obstetrics and Gynaecology, University of Melbourne, Parkville, Victoria Australia; 3 Murdoch Children’s Research Institute, Parkville, Victoria Australia; 4 Monash Newborn, Monash Children’s Hospital, Melbourne, Australia; 5 Department of Paediatrics, Monash University, Melbourne, Australia; 6 Department of Paediatrics, University of Melbourne, Melbourne, Australia

## Section 1: Administrative information

### 1a. Trial title

A multicentre, randomised trial of stabilisation with nasal high flow during neonatal endotracheal intubation: The SHINE Trial

### 1b. Trial registration

ANZCTR registration number ACTRN126128001498280

https://www.anzctr.org.au/Trial/Registration/TrialReview.aspx?id=375880&isReview=true

### 2. SAP version

Version 1 (dated 11 January 2021)

Version 2 (dated 18 June 2021)

### 3. Protocol version

This document is based on information contained in the study protocol of the SHINE Trial version 6 (dated 13 October 2020).

### 4. SAP revisions

The manuscript was initially submitted to Trials on April 1, 2021, prior to completion of primary data collection. Version 2 (dated 18 June 2021) made amendments following reviewer comments. These amendments were made prior to any data analysis occurring.

### 5. Roles and responsibilities

Trial Steering Committee

*Primary Investigator*

Dr Kate Hodgson

Newborn Research Centre, Royal Women’s Hospital,

Level 7, 20 Flemington Rd Parkville, Victoria, 3052, Australia

Kate.Hodgson@thewomens.org.au

+ 61407567360

*Chief Investigators*

A/Prof Brett Manley, The Royal Women’s Hospital, Parkville, Victoria

Dr Omar Kamlin, Royal Women’s Hospital, Parkville, Victoria

A/Prof Louise Owen, Royal Women’s Hospital, Parkville, Victoria

Prof Peter Davis, The Royal Women’s Hospital, Parkville, Victoria

*Trial Statistician*

A/Prof Susan Donath, Murdoch Children’s Research Institute, Parkville, Victoria

*Co-investigators*

Dr Calum Roberts, Monash Heath, Victoria

Dr Sophie Newman, Royal Women’s Hospital, Parkville, Victoria

Emily Twitchell, Royal Women’s Hospital, Parkville, Victoria

### 6. Signatures

This document outlines the proposed statistical analysis plan for the SHINE trial. It was prepared and approved by the SHINE Trial Steering Committee.

Approval:

**Table:** 

	
Dr Kate Hodgson	A/Prof Susan Donath
Principal Investigator	Trial Statistician
Date: 18/6/21	Date: 18/6/21

## Section 2: Introduction

### 7. Background and rationale for trial

Endotracheal intubation is an essential but potentially destabilising procedure for neonates. With an increased focus on avoiding mechanical ventilation, particularly in preterm infants, there are fewer opportunities for clinicians to gain proficiency in this important emergency skill. Rates of successful intubation at the first attempt are relatively low, and adverse event rates including desaturation and bradycardia are high, when compared with intubations in paediatric and adult populations. Interventions to improve operator success and patient stability during neonatal endotracheal intubation are needed. Using nasal high flow therapy during apnoea extends the safe apnoeica time of adults undergoing upper airway surgery and during endotracheal intubation [1]. This technique is untested in neonates.

The SHINE (Stabilisation with nasal High flow during Intubation of NEonates) trial is a multicentre, randomised controlled trial comparing the use of nasal high flow (nHF) during neonatal intubation with standard care (no nHF). Intubations are randomised individually and stratified by site, use of premedications, and postmenstrual age of the infant (≤ 28 weeks’ gestation; > 28 weeks’ gestation). The primary outcome is the incidence of successful intubation on the first attempt without physiological instability of the infant. Physiological instability is defined as an absolute decrease in peripheral oxygen saturation > 20% from pre-intubation baseline and/or bradycardia (< 100 beats per minute).

#### Research question

In neonates undergoing emergent or elective (with premedication) endotracheal intubation, does the use of nHF during laryngoscopy increase the likelihood of successful intubation on the first attempt without physiological instability, compared with no nHF?

### 8. Objectives

The primary objective of the SHINE trial is to investigate the efficacy of nHF in improving first attempt intubation success without physiological instability in neonates.

## Section 3: Study methods

### 9. Trial design

The SHINE trial is a multicentre, unblinded, randomised controlled trial investigating the efficacy of nHF to improve success and stability during neonatal endotracheal intubation. Intubations performed in the delivery room (DR) or neonatal intensive care unit (NICU) will be randomised, with a 1:1 ratio.

Infants will receive either:

1. Nasal HF during the endotracheal intubation attempt, or
2. Standard care (no nHF during the endotracheal intubation attempt)

Full explanation of the trial design is included in the trial protocol [2].

#### Study protocol development and conduct

The SHINE trial was registered with the Australian and New Zealand Clinical Trials Registry (ACTRN126128001498280) on 6 September 2018. The trial was approved by the Human Research Ethics Committee of The Royal Women’s Hospital (Melbourne, Australia) on 8 November 2018 and by the Human Research Ethics Committee of Monash Health (Melbourne, Australia) on 1 March 2019.

The consent process involves written, prospective consent wherever possible from parents for inclusion of their infant in the study. However, in the event of an emergency intubation in the DR or within the first 24 h after admission to NICU, the study has approval to use a retrospective consent process at both study sites. The infant will be included in the study, then consent to continue (retrospective consent) will be sought from the parent or guardian as soon as possible after the procedure. This consent process was pursued due to the known safety and efficacy of nHF use in neonates and the lack of any anticipated risk compared with standard clinical practice.

An independent data and safety monitoring board (DSMB) is monitoring the study progress. The trial will be reported according to the Consolidated Standards of Reporting Trials (CONSORT) guidelines [3].

#### Outcomes

The primary outcome is the incidence of successful intubation at the first attempt without physiological instability.

Definitions and secondary outcomes are further outlined in Section 6 and in the trial protocol [2].

### 10. Randomisation

Each intubation episode is randomly allocated in a 1:1 ratio to either nHF or control, stratified by:

1. Centre (Royal Women’s Hospital or Monash Newborn),
2. Postmenstrual age (≤ 28 weeks' gestation; > 28 weeks' gestation)
3. Use of premedication for intubation.

The randomisation sequence uses random permuted blocks with varying block sizes. To enable rapid randomisation following the decision to intubate by the clinical team, the randomisation is performed at the cot-side using a smartphone or computer with online access to the Research Electronic Data Capture (REDCap) [4] randomisation tool. Randomisation is web-based, using a password-protected, secure sockets layer (SSL)-encrypted website. The randomisation sequence was developed by an independent statistician at the Murdoch Children’s Research Institute, Melbourne, Australia. The group allocations are unblinded, due to the nature of the intervention. Intubation episodes, rather than infants, are randomised. An infant who has previously had an intubation episode randomised within the study can have subsequent intubation episodes randomised if (1) the premedication randomisation stratum differs between the studied intubations or (2) there is at least 1 week between the studied intubations for intubations where premedications are used.

### 11. Sample size

The sample size of 246 intubation episodes is based on a previous study [5], which examined 206 neonatal intubations by junior medical staff. This study found a 29% successful intubation rate at the first attempt without peripheral oxygen desaturation > 20% or bradycardia < 100 beats per minute. With a power of 90% to detect an increase in the incidence of successful intubation without physiological instability from 30 to 50%, and two-sided alpha 0.05, at least 123 intubation episodes in each group (246 total) are required.

### 12. Framework

The SHINE trial is investigating the superiority of nHF, compared with standard ca

re (no nHF) for the primary outcome. Secondary outcomes will also be compared using a superiority framework.

### 13. Statistical interim analysis and stopping guidance

An external DSMB has been convened and is chaired by Dr Chris McKinlay (Liggins Institute, University of Auckland, New Zealand). The DSMB consists of two consultant neonatologists (Dr Chris McKinlay and Dr Peter Dargaville) and an independent statistician (Dr Myra McGuinness). The terms of reference of the DSMB were outlined in the SHINE trial DSMB charter (version 4, 25 June 2019) and ratified by the Trial Steering Committee and all members of the DSMB during the first DSMB meeting.

Safety analyses, including of pre-defined significant adverse events (SAEs), were planned and performed after recruitment of:

- 60 patients (~ 25% total)
- 125 patients (~ 50% total) and
- 180 patients (~ 75% total).

The defined SAEs were:

- Incidence of pneumothorax within 72 h after randomisation, diagnosed either by transillumination of the chest and/or by chest X-ray
- Incidence of cardiac compressions and/or adrenaline administration within 1 h after the first intubation attempt
- Death within 72 h after randomisation

After 125 patients were recruited (~ 50% total), an interim efficacy analysis was undertaken, comparing the two treatment groups (blinded) for the primary endpoint and its components:

- Successful intubation on the first attempt without desaturation > 20% from baseline, or bradycardia < 100 bpm
- Successful intubation on the first attempt
- Desaturation > 20% from baseline
- Bradycardia < 100 bpm

The information was presented by pseudo-labelled treatment arm (e.g. ‘A’ and ‘B’); the key to identify the treatment arms was able to be supplied by the independent statistician if requested by the DSMB.

As per the DSMB charter, the DSMB could recommend stopping the trial on the basis of *safety* using clinical judgement informed by statistical comparison of adverse event rates. Accumulating signals of harm did not necessarily require statistically significant differences to warrant an alert and recommendation from the DSMB. The DSMB were also able to consider recommending stopping the trial if there was a very strong statistically significant difference (*p <* 0.001) in the primary outcome between groups at the interim efficacy analysis. There was no planned adjustment of the significance level due to interim analysis.

At each time-point, the DSMB recommended continuation of the trial, with an unchanged protocol.

### 14. Timing of final analysis

Final analysis will be conducted after data entry is completed and the database cleaned and closed.

#### Data collection and management

Demographic data are collected on paper Case Report Forms, or by directly inputting data into the REDCap [4] database, by investigators at the recruiting hospitals. Each intubation episode is video recorded using a GoPro camera (GoPro, San Mateo, California), placed in a location that provides a clear overhead view of the intubation procedure, the infant’s face and the Masimo (Irvine, CA, USA) pulse oximeter. Each video recording is reviewed by an investigator to determine primary and secondary outcomes. The primary outcome is also recorded on the Case Report Form in real time, in case of video failure. Outcomes are then entered into an secure, password-protected, online electronic database (REDCap [4]) by an investigator at each hospital.

After data entry, records were reviewed for missing data. Requests for addition of missing data or clarification were resolved by an investigator at each site.

All data will be checked and cleaned by the trial statistician, A/Prof Susan Donath, prior to analysis.

### 15. Timing of outcome assessments

The primary outcome is successful intubation on the first attempt, without physiological instability. The first intubation attempt is defined as the insertion and removal of laryngoscope beyond the baby’s lips.

The secondary outcomes are measured during the intubation episode (all intubation attempts for that infant) and up to 72 h after the intubation episode (for the pre-defined SAEs of pneumothorax, cardiac compressions and/or adrenaline administration, death).

## Section 4: Statistical principles

Overall principles

Data analysis will include all outcome data for all randomised intubation episodes. Analysis will start once all primary and secondary outcome are available, missing data has been sought, the database has been cleaned and locked, and the SAP has been submitted for publication.

### 17. Adjustment for multiplicity

All secondary outcomes will be reported as point estimates with unadjusted 95% confidence intervals only. There will be no adjustment for multiplicity.

### 18. Confidence intervals to be reported

For all outcomes, 95% confidence intervals will be presented.

### 19. Adherence and protocol deviations

### 20. Analysis population

The randomised population will comprise at least 246 intubation episodes, with infants randomised to either nHF or control. The analysis population will be created by removing the infants who met post-randomisation exclusion criteria from the randomised population, as outlined below.

On October 29, 2020, the Trial Steering Committee sought advice from the independent DSMB regarding post-randomisation exclusion criteria, with three deidentified randomisation episodes presented for discussion. These DSMB were blinded to trial data regarding the treatment arm and outcomes of the infants discussed.

Based on advice from the DSMB, the Trial Steering Committee reached consensus agreement regarding the following criteria for post-randomisation exclusions on 19 February 2021.

1. Randomised in error (patient was not intubated)
2. Failure to meet inclusion criteria
3. Meeting exclusion criteria at the time of randomisation (e.g. bradycardia, abdominal wall defect)
4. Parental withdrawal of consent
5. Parental consent declined in retrospective consent group

Therefore, the intention-to-treat (ITT) population will include all randomised infants and intubation episodes, regardless of exposure to the allocated treatment or adherence to the trial protocol, excluding the intubations which meet the post-randomisation exclusion criteria described above.

#### Per-protocol analysis

We will undertake a per protocol analysis for the primary outcome if there are infants in the control group who received nHF or other apnoeic oxygenation during intubation or infants in the nHF group who never received the intervention. The following will not be deemed protocol violations: high flow prongs are placed and then dislodge or mechanical failure of machine.

## Section 5: Trial population

### 21. Screening data

**Fig. 1:**
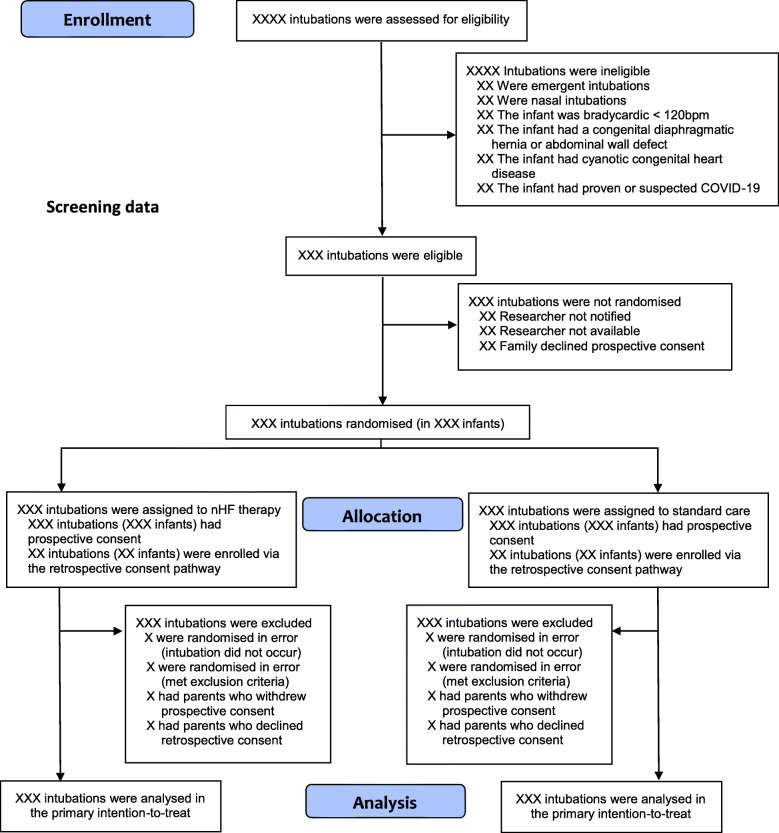
Consolidated Standards of Reporting Trials (CONSORT) 2010 flow diagram

All intubation episodes in both centres will be assessed for eligibility for inclusion in the trial. The CONSORT flow diagram in Fig. 1 will be used to detail enrolment, randomisation, treatment allocation, follow-up, and analysis.

### 22. Eligibility criteria

Any patient undergoing endotracheal intubation in the DR or NICU is eligible for inclusion. Specific inclusion and exclusion criteria are outlined in the protocol [2].

### 23. CONSORT diagram

Please see Fig. 1.

### 24. Withdrawal/follow-up

Infants where prospective consent is withdrawn, or retrospective consent is not gained, will be treated as post-randomisation exclusions (Fig. 1).

### 25. Baseline patient characteristics at randomisation

The following baseline characteristics will be summarised (see Table 1):

- Mothers:
- Mode of delivery: vaginal delivery, caesarean section under spinal anaesthesia, caesarean section under general anaesthesia: number (%)
- Infants:
- Gestational age (weeks): mean (standard deviation, SD)
- Birth weight (grams): mean (SD)
- Age at randomisation (hours): mean (SD)
- Corrected gestational age at randomisation (weeks): mean (SD)
- Weight at randomisation (grams): mean (SD)
- Male: number (%)
- Multiple birth: number (%)
- Apgar score at 5 min: median (interquartile range)
- Respiratory support prior to randomisation (no support, nHF, continuous positive airway pressure, intermittent positive pressure ventilation): number (%)
- Fraction of inspired oxygen prior to randomisation: (mean, SD)

**Table 1:** Baseline characteristics at time of randomisation

Characteristic	Nasal HF group (*n =* XXX)	Control group (*n =* XXX)
Mothers		
Mode of delivery—no. (%)		
Vaginal delivery	XX (%)	XX (%)
Caesarean section under spinal anaesthesia	XX (%)	XX (%)
Caesarean section under general anaesthesia	XX (%)	XX (%)
Infants		
Gestational age at birth—weeks	Mean (SD)	Mean (SD)
≤ 28 weeks	XX (%)	XX (%)
> 28 weeks	XX (%)	XX (%)
Birth weight—grams	Mean (SD)	Mean (SD)
Age at randomisation—hours	Mean (SD)	Mean (SD)
Corrected GA at randomisation—weeks	Mean (SD)	Mean (SD)
Weight at randomisation—grams	Mean (SD)	Mean (SD)
Male—no. (%)	XX (%)	XX (%)
Multiple birth—no. (%)	XX (%)	XX (%)
Apgar score at 5 min	Mean (SD)	Mean (SD)
Respiratory support prior to randomisation		
Nasal high flow	XX (%)	XX (%)
Continuous positive airway pressure	XX (%)	XX (%)
Intermittent positive pressure ventilation (IPPV) (via face mask, does not include IPPV following premedication)	XX (%)	XX (%)
IPPV via endotracheal tube	XX (%)	XX (%)
Low flow oxygen	XX (%)	XX (%)
No respiratory support	XX (%)	XX (%)
Fraction of inspired oxygen prior to randomisation	Mean (SD)	Mean (SD)
**Intubation characteristic**		
Primary reason for intubation—no. (%)		
Hypoxia	XX (%)	XX (%)
Hypercarbia	XX (%)	XX (%)
Apnoea	XX (%)	XX (%)
Resuscitation	XX (%)	XX (%)
Other	XX (%)	XX (%)
Use of premedication—no. (%)		
Premedication	XX (%)	XX (%)
No premedication	XX (%)	XX (%)
First intubation attempt operator—no. (%)		
Resident/registrar/neonatal nurse practitioner	XX (%)	XX (%)
Fellow/consultant	XX (%)	XX (%)
Experience of operator (number of previous intubations)—no. (%)		
< 20 previous intubations	XX (%)	XX (%)
≥ 20 previous intubations	XX (%)	XX (%)

N.B. Baseline demographic characteristics are for all intubation episodes

## Section 6: Analysis

### 26. Outcome definitions

For all incidence outcomes, incidence is the proportion of first intubation attempts in which that outcome occurred.

#### Primary outcome

The primary outcome is incidence of successful intubation at the first attempt without physiological instability, i.e. the proportion of first intubation attempts in which successful intubation without physiological instability occurs (Table 2).

**Table 2:** Primary outcome and components

Outcome	Nasal HF group (*n =* XXX)	Control group (*n =* XXX)	Risk difference (95% CI)
Intention-to-treat analysis			
Successful first attempt intubation without physiological instability	XX (%)	XX (%)	
≤ 28 weeks’ GA	XX (%)	XX (%)	
> 28 weeks’ GA	XX (%)	XX (%)	
Premedication use	XX (%)	XX (%)	
No premedication use	XX (%)	XX (%)	
Inexperienced operator (< 20 previous intubations	XX (%)	XX (%)	
Experienced operator (≥ 20 previous intubations)	XX (%)	XX (%)	
Successful first attempt intubation	XX (%)	XX (%)	
Desaturation (SpO_2_ > 20% from baseline) during the first intubation attempt	XX (%)	XX (%)	
Bradycardia (HR < 100 bpm) during the first intubation attempt	XX (%)	XX (%)	

#### Definitions

- Intubation attempt: the insertion of the laryngoscope blade beyond the infant’s lips.
- Intubation duration: the time from the insertion of the laryngoscope blade beyond the infant’s lips until the removal of the laryngoscope blade from the infant’s mouth.
- Successful intubation: the completion of the intubation attempt with correct positioning of the endotracheal tube confirmed by detection of expired carbon dioxide on a colorimetric detector.
- Physiological instability: the incidence (any duration) of an absolute decrease in peripheral oxygen saturation (SpO_2_) > 20% from baseline (immediately prior to the intubation attempt), and/or bradycardia (heart rate < 100 beats per minute, bpm), during the first intubation attempt.

#### Secondary outcomes

- Incidence of successful intubation on the first intubation attempt.
- Incidence of desaturation (absolute decrease in SpO_2_ > 20% from baseline) *or* bradycardia (heart rate < 100 bpm) during the first intubation attempt.
- In first intubation attempts where desaturation (absolute decrease in SpO_2_ > 20% from baseline) occurs, time to desaturation during the first intubation attempt in seconds.
- In first intubation attempts where bradycardia (heart rate < 100 bpm) occurs, time to bradycardia during the first intubation attempt in seconds.
- In first intubation attempts where desaturation (absolute decrease in SpO_2_ > 20% from baseline) occurs, duration of desaturation during first intubation attempt in seconds.
- In first intubation attempts where bradycardia (heart rate < 100 bpm) occurs, duration of bradycardia during first intubation attempt in seconds.
- Median SpO_2_ during first intubation attempt.
- Median heart rate during first intubation attempt.
- In first intubation attempts where SpO_2_ > 97% occurs, duration of SpO_2_ > 97% during first intubation attempt, in seconds.
- Number of intubation attempts.
- Duration of all intubation attempts (successful and unsuccessful), in seconds.
- Incidence of cardiac compressions and/or adrenaline administration within 1 h after the first intubation attempt.
- Incidence of pneumothorax within 72 h after randomisation, diagnosed either by transillumination of the chest and/or by chest X-ray.
- Incidence of pneumothorax requiring drainage (via needle thoracocentesis or insertion of an intercostal catheter) within 72 h after randomisation.
- Death within 72 h after randomisation.

### 27. Analysis methods

For all outcomes, the difference between the 2 treatment groups will be estimated using multivariable regression, with the outcome as the dependent variable, the group allocation as the predictor, and the stratification factors used during randomisation (gestational age group, premedication use and trial centre) as covariates.

For binary outcome variables, including the primary outcome, the number and percentage of intubation attempts with the outcome will be presented separately for the 2 treatment groups. Binary regression (fitting a generalised linear model) will be used to estimate the difference between the treatment groups. The results will be reported as the difference in risk between the 2 treatment groups, with the 95% CI for the risk difference.

For continuous outcomes, the distribution of each outcome will be assessed visually using graphical methods (histogram and dotplot). If the distribution is considered to be so skewed that the mean is an inappropriate summary measure, the outcome will be summarised using the median, otherwise the outcome will be summarised by the mean.

Where the summary measure is the mean, the mean and SD will be presented separately for the 2 treatment groups. Linear regression will be used to estimate the difference between the treatment groups. The results will be reported as the difference of means between the 2 treatment groups, with the 95% CI for the difference of means.

Where the summary measure is the median, the median and IQR will be presented separately for the 2 treatment groups. Quantile regression will be used to estimate the difference between the treatment groups. The results will be reported as the difference of medians between the 2 treatment groups, with the 95% CI for the difference of medians.

#### Analyses- primary outcome

The primary analysis will be a modified intention to treat analysis, using the exclusion criteria outlined above. The primary outcome is a binary outcome, so as described above, the number and percentage of intubation attempts where there was successful intubation at the first attempt without physiological instability will be presented separately for the 2 treatment groups. Binary regression (a generalised linear multivariable model, with the primary outcome as the dependent variable, the group allocation as the predictor, and the stratification factors used during randomisation as covariates) will be used to estimate the difference between the treatment groups. The results will be reported as the difference in risk between the 2 treatment groups, with the 95% CI for the risk difference.

#### Primary outcome—sensitivity analyses

As some infants will be randomised more than once, the difference in risk of the primary outcome between the 2 treatment groups, with the 95% CI for the risk difference, adjusted for repeated measures, will also be reported. The model used for this analysis will be a multivariable binary regression as described above, with standard errors estimated allowing for intragroup correlation (using the vce(cluster) option in Stata).

If an imbalance in demographics known to affect intubation success (e.g. postmenstrual age, weight, videolaryngoscope use, operator experience) is detected, a further sensitivity analysis adjusting for the relevant demographics will be conducted for the primary outcome.

#### Primary outcome—subgroup analyses

We will perform pre-specified subgroup analyses for the primary outcome. The prespecified subgroup analyses included in the protocol are:

1. Postmenstrual age (≤ 28 weeks' gestation; > 28 weeks' gestation)
2. Use of premedication for intubation (yes or no)

In addition, we will perform a pre-specified subgroup analysis for:

3. Operator experience (inexperienced, < 20 previous intubations or experienced, ≥ 20 previous intubations)

This subgroup analysis has been specified following the original trial protocol publication, but prior to submission of this statistical analysis plan or performing any data analysis.

The proceduralists are the clinical staff employed at the two centres:

- The Royal Women’s Hospital (13 consultants, 10 fellows, 17 residents, 2 nurse practitioners
- Monash Newborn (13 consultants, 12 fellows, 13 residents, 4 nurse practitioners)

As the study is not powered for subgroup analysis, these analyses are considered exploratory. Three additional adjusted models will be estimated to explore potential heterogeneity of the effect of the intervention. Each model will include as covariates the stratification factors used in randomisation and an interaction term estimating the interaction between the intervention and the subgroup variable (listed above). Specific subgroup estimates and confidence intervals will be presented obtained from the adjusted model. If there is no evidence of interaction (*p* > 0.05), any differences between subgroups will be regarded as due to chance.

#### Analyses: secondary outcomes

Secondary outcomes will be analysed as described above. The results will be presented as outlined in Tables 3 and 1 (components of the primary outcome).

**Table 3:** Secondary outcomes

Outcome	Nasal HF group (*n =* XXX)	Control group (*n =* XXX)	Risk difference or difference of means or difference of medians (95% CI)
Time to desaturation—seconds^a^	Mean (SD) or median (IQR)	Mean (SD) or median (IQR)	
Duration of desaturation—seconds^a^	Mean (SD) or median (IQR)	Mean (SD) or median (IQR)	
Median SpO_2_^a^	Mean (SD) or median (IQR)	Mean (SD) or median (IQR)	
Bradycardia (HR < 100 bpm)^a^	XX (%)	XX (%)	
Time to bradycardia—seconds^a^	Mean (SD) or median (IQR)	Mean (SD) or median (IQR)	
Duration of bradycardia—seconds^a^	Mean (SD) or median (IQR)	Mean (SD) or median (IQR)	
Median HR^a^	Mean (SD) or median (IQR)	Mean (SD) or median (IQR)	
Number of intubation attempts	Mean (SD) or median (IQR)	Mean (SD) or median (IQR)	
Total duration of all intubation attempts (successful and unsuccessful)—seconds^c^	Mean (SD) or median (IQR)	Mean (SD) or median (IQR)	
CPR and/or adrenaline administration within 1 h of intubation attempt^b^	XX (%)	XX (%)	
Pneumothorax diagnosed within 72 h after randomisation^b^	XX (%)	XX (%)	
Any	XX (%)	XX (%)	
Requiring drainage with needle thoracocentesis or intercostal catheter	XX (%)	XX (%)	
Death within 72 h after randomisation^b^	XX (%)	XX (%)	

^a^During first intubation attempt

^b^Specified as serious adverse events in trial

^c^Sum of each separate intubation attempt

*IQR* interquartile range, *SpO*_*2*_ peripheral oxygen saturation, *HR* heart rate, *Bpm* beats per minute, *CPR* cardiopulmonary resuscitation

There will be no subgroup analyses performed for secondary outcomes.

### 28. Missing data

Every attempt will be undertaken to retrieve missing data. The primary outcome is recorded on a paper CRF by the investigator at the cot-side, to provide a backup to the video recording and time stamped downloadable oximetry recording. We therefore expect there to be very few instances in which the primary outcome cannot be determined and therefore do not anticipate needing to use multiple imputation to deal with missing data. Imputation will not be used for missing physiological data, for example in the event of loss of pulse oximetry signal or failure of video recording.

Multiple imputation or inverse probability case weights may be used to deal with missing data.

### 29. Additional analyses

Subsequent analyses that are not specified in the protocol may be performed if requested by journal editors or reviewers. These will be performed consistently with the principles of this analysis plan, as far as possible. Subsequent analyses of a more exploratory nature will not be bound by this strategy, but are expected to follow the broad principles described.

There will be graphical displays of results to present data.

Other additional analyses to be analysed and reported subsequent to the main trial include an additional sub-study will examine Near InfraRed Spectroscopy (NIRS) in a subset of babies undergoing randomisation in the trial. These data will be analysed separately and submitted for publication separately.

### 30. Harms

Incidence of the following serious adverse events will be compared between groups:

1. Incidence of pneumothorax within 72 h after randomisation, diagnosed by either transillumination of the chest and/or by chest X-ray
2. Incidence of pneumothorax requiring drainage (via needle thoracocentesis or insertion of an intercostal catheter) within 72 h after randomisation
3. Incidence of cardiac compressions and/or adrenaline administration within 1 h after the first intubation attempt
4. Death within 72 h after randomisation

These outcomes will be reported with 95% CI, without adjustment for multiplicity, given that type I error rates larger than 0.05 may be important. If journal editors or reviewers request it, *P* values may be reported for the comparisons of adverse events between treatment groups.

### 31. Statistical software

Data will be exported from the study database to STATA (StataCorp. 2019. *Stata Statistical Software: Release 16*. College Station, TX: StataCorp LLC) for analysis.

## Data Availability

The datasets during and/or analysed during the current study will be available from the corresponding author on reasonable request.
